# Lost and found: applying network analysis to public health contact tracing for HIV

**DOI:** 10.1007/s41109-021-00355-w

**Published:** 2021-02-17

**Authors:** Dana K. Pasquale, Irene A. Doherty, Peter A. Leone, Ann M. Dennis, Erika Samoff, Constance S. Jones, John Barnhart, William C. Miller

**Affiliations:** 1 Department of Sociology, Duke University, 417 Chapel Drive, 276 Soc/Psych Building, Box 90088, Durham, NC 27708-0088 USA; 2 Department of Epidemiology, Gillings School of Global Public Health, University of North Carolina At Chapel Hill, Chapel Hill, NC USA; 3 RTI International, Research Triangle Park, Durham, NC USA; 4 The Julius L. Chambers Biomedical Biotechnology Research Institute, North Carolina Central University, Durham, NC USA; 5 Division of Infectious Diseases, School of Medicine, University of North Carolina at Chapel Hill, Chapel Hill, NC USA; 6 NC Department of Health and Human Services Communicable Disease Branch, Raleigh, NC USA; 7 Division of Epidemiology, College of Public Health, The Ohio State University, Columbus, OH USA

**Keywords:** HIV-1, Sexual networks, Public health, Partner notification, Contact tracing, Syphilis, Epidemiology, North Carolina

## Abstract

Infectious disease surveillance is often case-based, focused on people diagnosed and their contacts in a predefined time window, and treated as independent across infections. Network analysis of partners and contacts joining multiple investigations and infections can reveal social or temporal trends, providing opportunities for epidemic control within broader networks. We constructed a sociosexual network of all HIV and early syphilis cases and contacts investigated among residents of 11 contiguous counties in North Carolina over a two-year period (2012–2013). We anchored the analysis on new HIV diagnoses (“indexes”), but also included nodes and edges from syphilis investigations that were within the same network component as any new HIV index. After adding syphilis investigations and deduplicating people included in multiple investigations (entity resolution), the final network comprised 1470 people: 569 HIV indexes, 700 contacts to HIV indexes who were not also new cases themselves, and 201 people who were either indexes or contacts in eligible syphilis investigations. Among HIV indexes, nearly half (48%; n = 273) had no located contacts during single-investigation contact tracing, though 25 (9%) of these were identified by other network members and thus not isolated in the final multiple investigation network. Constructing a sociosexual network from cases and contacts across multiple investigations mitigated some effects of unobserved partnerships underlying the HIV epidemic and demonstrated the HIV and syphilis overlap in these networks.

## Introduction

Several aspects of the network of relationships among which human immunodeficiency virus (HIV) diffuses can help guide intervention, including discerning the relationship ties (edges) along which the infection diffuses (Doherty et al. 2005), ascertaining mixing patterns and risk assortativity (Doherty et al. 2009, 2011; Schneider et al. 2013), and assessing the overlap of HIV and other sexually transmitted infections (STI) circulating within a socio-sexual network. HIV and syphilis, a bacterial infection, are both epidemics and in the southeastern United States (US).

Though HIV can be spread through behaviors such as sharing needles and syphilis can be transmitted during childbirth, in this region both share a population at risk and are primarily sexually transmitted. A single sexual act can transmit both infections. They are biologically synergistic, where being infected with one increases the possibility of acquiring the other infection (Augenbraun and McCormack 1994; Chesson et al. 1999; Buchacz et al. 2004; Karp et al. 2009; Mayer and Venkatesh 2011), and thus represent a complex public health problem (Fujimoto et al. 2018). Indeed, prior work has demonstrated that overlap of HIV and syphilis in sexual network components increases transmission risk (Fujimoto et al. 2018; Doherty et al. 2011; Cope et al. 2014).

HIV and syphilis are *reportable diseases* in all 50 states plus the District of Columbia (Institute of Medicine (US) Committee on the Ryan White CARE Act 2004), meaning that any healthcare provider or laboratory must report diagnoses to their governing public health body. Trained public health personnel meet with “index cases”—people who are newly diagnosed—to perform *partner notification interviews* and *contact tracing* for each new case of HIV and/or syphilis reported. During partner notification interviews, risk behaviors of the newly diagnosed index case are assessed and the case is asked to disclose sexual, needle-sharing, or social contacts who would benefit from sexually transmitted infection testing so that the public health personnel can trace the contacts. During contract tracing, these personnel locate these contacts, inform them of their potential risk, offer testing, and assist with treatment appointments if necessary. Though these person-based data lend themselves to network construction, infectious disease surveillance often focuses on individual cases and contacts, treating infected people and their traced contacts cross-sectionally and independently across infections. Network analysis of partners and contacts across investigations—both within and across multiple infections—can reveal social or temporal trends and provide opportunities for broader epidemic control (Liljeros et al. 2003), particularly among synergistic infections such as HIV and syphilis where decreasing the incidence of one infection may also decrease the incidence of the other infection.

In North Carolina (NC), as in other parts of the US South, young Black men disproportionately bear the burden of both HIV and syphilis (Sena et al. 2008; Centers for Disease Control and Prevention 2016, 2019). In North Carolina, Black men age 13 years and older make up less than one-quarter of the population (North Carolina HIV/STD/Hepatitis Surveillance Unit 2020b) yet accounted for approximately half (674/1383) (North Carolina HIV/STD/Hepatitis Surveillance Unit 2020a) of all new HIV diagnoses in 2019. As these young men are also less likely to have regular healthcare access (Krawczyk et al. 2006; Napravnik et al. 2006), public health contact tracing activities to locate, test, and treat them are critical. However, this mission is hampered by mistrust and social/structural issues which lead to incomplete contact ascertainment.

Contact tracing is name-based and conducted in NC by public health professionals called disease intervention specialists (DIS). DIS attempted to interview all persons newly diagnosed with HIV and/or early syphilis (“index cases”) in the state. In these interviews, DIS elicit sexual partners, injecting drug use partners, and social contacts thought by the interviewee to possibly be at high risk of HIV exposure (Centers for Disease Control and Prevention 2008). The period of interest for which DIS elicit contacts during HIV investigations is the 12 months prior to diagnosis for established HIV cases; 6 months prior for persons thought to be recently infected based on acute viral illness or recent negative HIV test; and 2 months prior for persons diagnosed during acute HIV infection (AHI) per 4th generation antibody or RNA test results (Centers for Disease Control and Prevention 2014). The period of interest for syphilis is 3 months prior for primary syphilis, 6 months prior for secondary syphilis, and 12 months prior for early latent syphilis (Centers for Disease Control and Prevention 2014). For both infections, DIS also elicit and trace social contacts at their discretion since there appears to be an overlap between social networks and sexual partners (Brenner et al. 2011), particularly among Black men who have sex with men (MSM) (Schneider et al. 2013; Tieu et al. 2015). Upon elicitation, DIS determine whether there is enough information on each contact to begin the process of attempting to trace them (“investigation”); contacts without enough locating information are deemed “marginal” partners and not traced, though marginal partners can revert to investigated partners if more locating information is uncovered. Contacts with sufficient locating information are “initiated” for tracing and testing. DIS then investigate the initiated contacts, which includes a records search in the database and/or an attempt to locate the contact for testing and interview. All cooperation with contact tracing is voluntary.

We sought to augment information gleaned from individual HIV investigations in central NC by combining multiple HIV investigations and adding information from early syphilis investigations into a single network. We then described where and how consideration of the broader network added context through understanding component composition that would not be known by treating each case independently. Finally, we assessed the overlap between HIV and syphilis circulating among network components constructed from disclosed contacts.

## Methods

### Study population, setting, and data

**Fig. 1:**
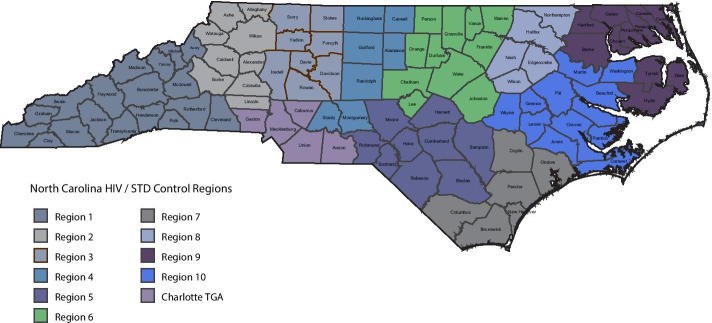
North Carolina Prevention and Care Services Regions. Legend: Map showing the North Carolina Regions for Prevention and Care Services. We abstracted HIV and syphilis cases diagnosed during 2012–2013 from among residents of the 11 contiguous counties of Region 6 (green counties)

NC is divided into 10 regions for HIV and sexually transmitted disease (STD) control activities. Region 6 in north central NC (Fig. 1) comprises seven metropolitan and four non-metropolitan counties based on 2013 US Department of Agriculture rural–urban continuum codes (USDA Economic Research Service 2013), with a total population in 2013 of ~ 1.9 million persons including ~ 8,700 persons living with HIV (North Carolina HIV/STD/Hepatitis Surveillance Unit 2015b). The rate of new HIV diagnoses in Region 6 was 16.3 per 100,000 population in 2013, corresponding to 315 new diagnoses (North Carolina HIV/STD/Hepatitis Surveillance Unit 2015b). Black men who have sex with men (MSM) were disproportionately affected, accounting for nearly half of all HIV diagnoses among men (North Carolina HIV/STD/Hepatitis Surveillance Unit 2015a).

We abstracted infectious disease surveillance and linked index-and-contact public health contact tracing data elicited during partner notification for HIV and syphilis diagnoses made among residents aged ≥ 14 years of the 11 contiguous counties in Region 6 over a two-year period (2012–2013). We matched the named cases and contacts across all contact tracing investigations conducted in these counties during the two-year period. Some contacts were diagnosed as part of contact tracing activities (i.e., as contacts of newly diagnosed cases) and were then also considered to be an index if they were diagnosed during 2012–2013 while residing in one of the 11 counties under study. As entering these sociosexual networks with high HIV prevalence is associated with future HIV acquisition among Black MSM (Hurt et al. 2012), we also checked for new HIV diagnoses made through 2018 among network members who were not known to be HIV-positive during the 2012–2013 study period.

### Sociosexual network construction

Contact tracing and partner notification data collected by DIS during HIV and syphilis investigations were used to create an undirected sociosexual network of disclosed ties using the igraph (Csárdi and Nepusz 2006) package in R (R Core Team. R 2015). Nodal attributes collected via self-report included demographic characteristics (ethnicity/race, five-year age category, gender/sexual orientation, county of residence), risk behaviors (drug use, condom use, sexual relationships with people known to be HIV-positive, partnership dynamics), and HIV and STD testing and infection history. Edge attributes included the type of relationship (sexual, social, needle-sharing) and the infection being investigated.

We created the edgelist from relationships elicited across multiple HIV and syphilis case investigations. All network members were de-identified after edgelist construction to preserve confidentiality. All contacts disclosed by newly diagnosed HIV or syphilis cases in this geographic area were used to construct the edgelist and are represented in the network, even if the relationships were not concurrent. We have several reasons for constructing the network in this way: young men who enter these components frequently remain linked into them and go on to acquire HIV at higher rates (Hurt et al. 2012); both HIV and syphilis circulating among the same group carries a potential risk for both infections even if partnerships are not concurrent; and HIV is a lifelong infection. While transmission can be prevented through condom use and antiretroviral therapy to reduce viral load and thus infectiousness, nearly 1 in 3 HIV indexes in this set (n = 180) failed to achieve viral suppression within three years of diagnosis, with Black men less likely than men of other races, and another 11% (n = 65) had inconsistent viral suppression during the three-year post-diagnosis period. Thus, contacts which occurred after the HIV index’s diagnosis still represent a pathway to consider for infection diffusion given that at least one of the nodes would be HIV-positive and some of the components also had circulating syphilis.

We compiled the network from the edgelist. Our primary focus was HIV index case investigations; partnerships elicited during syphilis investigations in Region 6 during 2012–2013 were only included to better understand network connectivity among persons at risk of HIV. As such, syphilis network components that did not include at least one HIV case were excluded from the analysis network since they would not affect network metrics calculated with respect to HIV indexes. The University of North Carolina Biomedical Institutional Review Board approved the study.

### Descriptive network analyses

Improvements in network ascertainment and changes in connectivity were measured at each step of DIS partner services activities where a case might have no traced partners yet was identified by another network member. We compared network structures between indexes who disclosed contacts who were initiated for partner services to indexes who did not have any outgoing contacts initiated but who were identified by another network member and so were not isolated in the final observed network. We assessed how network construction can provide additional context to HIV investigations as measured by a decrease in the number of HIV indexes with no known contacts and reveal the overlap.

The network immediately surrounding each HIV index was treated as a local ego network and network characteristics and structures (*k*-cores, triangles) were calculated on the basis of index network position. We measured component sizes and distribution, component composition by sociodemographic characteristics and infection type, and bridging of HIV investigation edges by syphilis investigation edges determined from separate contact tracing efforts.

## Results

### Study population

During the two-year study period (2012–2013), 569 new HIV index diagnoses were reported among residents aged ≥ 14 years across the eleven Region 6 counties under study; these HIV indexes formed the core of the sociosexual network analyzed. Most were male (79%) and non-Hispanic Black (66%). Median age at diagnosis was 33 years (IQR: 24–45) (Table 1). Laboratory results indicated that 32 (6%) were acutely or recently infected with HIV and 144 (25%) had already progressed to acquired immunodeficiency syndrome (AIDS) by the time of diagnosis.

**Table 1:** Index HIV cases aged 14 years and older diagnosed 2012–2013 in NC HIV/STD Control Region 6 and their disclosed and traced first-degree contacts in the sociosexual network (N = 1269)

	Index (n = 569)		Contact (n = 700)	
	n	(%)	n	(%)
Gender				
Male	451	(79)	581	(83)
Female	114	(20)	98	(14)
Transgender (M to F)	4	(0.7)	0	–
Not indicated	0	–	21	(3)
Race/Ethnicity				
Non-Hispanic White	114	(20)	164	(23)
Non-Hispanic Black	378	(66)	459	(66)
Hispanic, any single race	58	(10)	33	(5)
Other or mixed race	19	(3)	31	(4)
Not indicated	0	–	13	(2)
Region of birth				
USA-50 states	530	(93)	344	(49)
Latin / South America, Caribbean (incl. US Territories)	24	(4)	6	(0.9)
Europe, Asia, Oceania	3	(0.5)	2	(0.3)
Africa	12	(2)	0	–
Not indicated	0	–	348	(50)
Marital status				
Currently married	39	(7)	44	(6)
Divorced / separated / widowed	20	(4)	9	(1)
Never married	413	(73)	413	(59)
Not indicated	97	(17)	234	(33)
County of residence				
Urban	437	(77)	–-	
Suburban	27	(5)	–-	
Rural	105	(18)	–-	
Age at index case's HIV diagnosis (years)^a^				
≤ 19	29	(5)	46	(7)
20–29	214	(38)	316	(45)
30–39	101	(18)	169	(24)
40–49	136	(24)	88	(13)
≥ 50	89	(16)	52	(7)
Not indicated	0	–-	29	(4)
Median (IQR)	33	(24–45)	28	(23–37)
HIV status				
Positive	569	(100)	221	(35)
Negative	–-		243	(32)
Unknown	–		236	(34)
Year of HIV diagnosis			n = 221	
< 2006	–		50	(23)
2006–2010	–		71	(32)
2011	–		21	(10)
2012	271	(48)	13	(6)
2013	298	(52)	7	(3)
≥ 2014	–		35	(16)
Not indicated	–		24	(11)
HIV stage at diagnosis^b^				
Acute/recent	32	(6)	–	
Chronic, non-AIDS	393	(69)	–	
Chronic, AIDS	144	(25)	–	

^a^Among partners, for earliest record associated with any index case

^b^Based upon laboratory results

### Contact tracing

DIS interviewed nearly all indexes (97%), although 26% indexes (n = 146) declined to discuss or provide names of contacts. The 74% who disclosed contacts (n = 423) reported a total of 1,850 sexual partners (median = 2 (IQR: 1–4), range 0–60), 130 social contacts (range 0–19), and 5 needle-sharing partners (range 0–3) in the 2, 6, or 12 months prior to diagnosis (depending on infection stage). Of the 1,850 sexual partners reported, 521 (28%) did not have enough locating information to initiate partner notification.

### Relationships with investigated contacts

DIS ultimately succeeded in documenting 845 relationships for notification and testing: 749 sexual partnerships, 92 social contacts, and 4 needle-sharing partnerships, representing 40%, 71%, and 80% of total partnerships/contacts reported, respectively. These relationships composed the sociosexual network edges. Indexes with at least one investigated contact had a median of 2 first-degree network contacts (IQR:1–3, range: 1–25). Most sexual partnerships were among people of the same race (78%), included at least one person of Black race (77%), and were between two men (72%). In half (51%) of sexual partnerships, partners were within five years of age of each other. Among the 749 sexual and 4 needle-sharing partners, 42% (319/753) were also HIV-infected including some who diagnosed as indexes themselves during the study period 2012–2013 (diagnosis year range 1995–2017, 46% diagnosed ≥ 6 months prior to index), 30% of contacts tested HIV-negative during the investigation, and 27% had unknown serostatus.

### Sociosexual network population

**Fig. 2:**
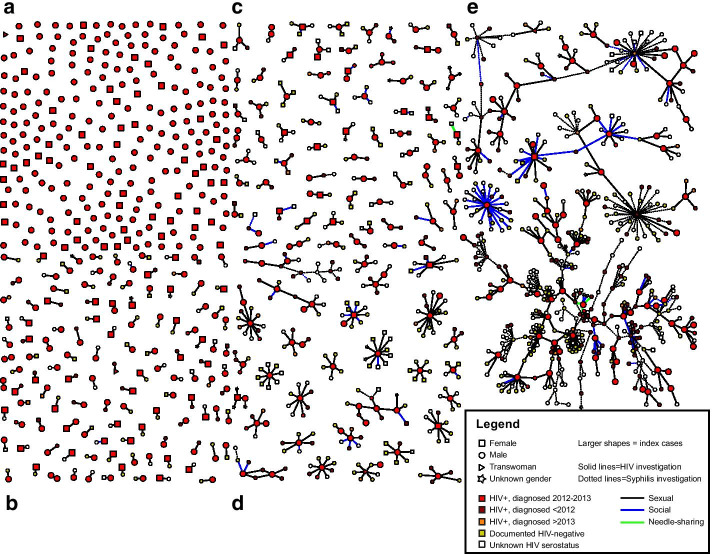

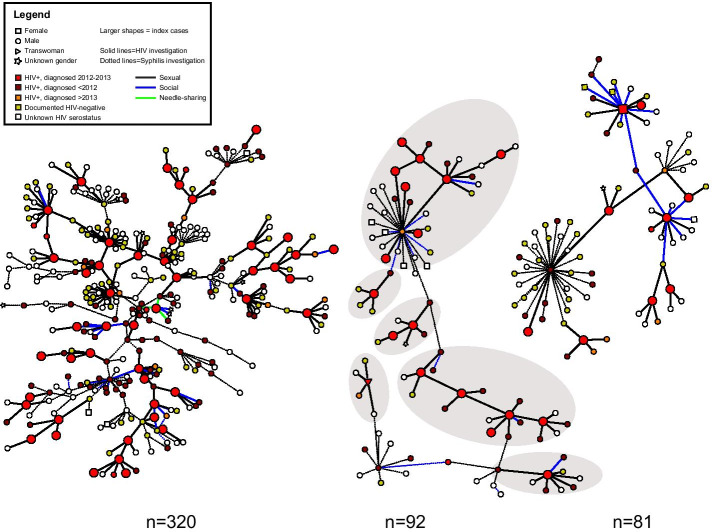
**a.** Sociosexual network (N = 1470). Sociosexual network showing 569 index cases newly diagnosed with HIV in the area around Raleigh, North Carolina, during 2012–2013. Total graph includes 1470 persons distributed in 468 network components. Graph shows gender (node shape), HIV status (node color), HIV index case status (node size), type of contact (edge color), and whether the contact was part of an HIV or syphilis investigation (edge thickness). Graph is loosely grouped by size of sociosexual network component: **a** isolates (n = 248 people), **b** dyads (n = 238 people distributed across 119 components), **c** components size 3–4 (n = 224 people distributed across 68 components), **d** components size 5–16 (n = 241 people distributed across 29 components), and e) components size 26, 81, 92, and 320 (n = 519 people distributed across 4 components). **b.** Three largest components of the sociosexual network (n = 493). Legend: Sociosexual network showing the three largest components representing 493/1,470 (34%) network members (from left to right, 320, 92, and 81 people, respectively). Graph shows gender (node shape), HIV status (node color), index case status (node size), type of contact (edge color), and whether the contact was part of an HIV or syphilis investigation (edge thickness). The middle component (n = 92 nodes) would have been observed as six smaller HIV investigation components (indicated by gray background) without inclusion of the syphilis investigation partnerships bridging the relationships elicited during the HIV investigations

All indexes and any contacts investigated were included in the network. After de-duplicating people who appeared in multiple investigations (entity resolution: 106/845 contacts were indexes themselves and the other 39 were contacts of > 1 index), 700 unique first-degree HIV contacts were added to the network in addition to the 569 indexes. After de-duplication, the syphilis investigations added 201 more people who were in the same component as at least one HIV index. The final total network size was 1470 persons (Fig. 2a) and included 569 newly-diagnosed HIV indexes plus 901 network members who were observed to be first-degree contacts of an HIV index (n = 700) or indirectly linked to an HIV index in the same sociosexual network component (n = 201), while not being HIV index cases themselves. Of these 901, 283 (31%) network members were HIV-positive (median diagnosis year 2009 (IQR: 2006–2012) excluding 80 with unknown diagnosis years), 272 (30%) were HIV negative based upon a test during the investigation period, and serostatus was unknown for the remaining 346 (38%) due to inability to locate the contact upon investigation or the contact refusing counseling and/or current testing upon location.

### Sociosexual network construction and composition

Despite interviewing 97% (n = 551) of HIV indexes, nearly half (48%, 273/569) had no located contacts during their individual contact tracing investigation. After adding syphilis investigation information and creating a network, 25 of 273 (9%) HIV indexes without any traced contacts were identified by other network members and thus were not isolated in the final observed network (n = 248 remained isolated with no located contacts).

**Fig. 3:**
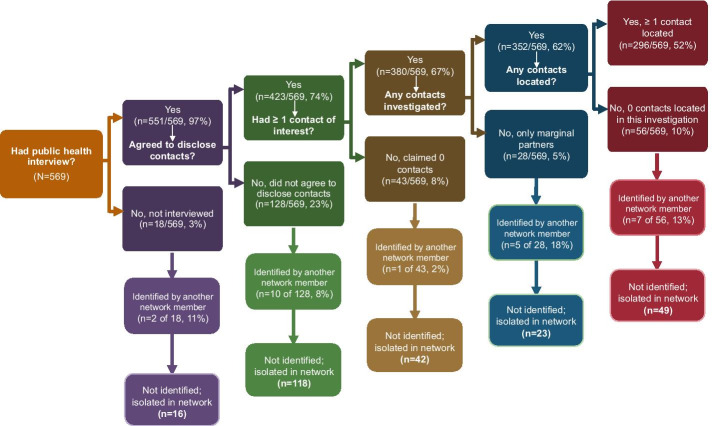
Public health interview continuum and network isolation. This figure shows the progress along the disease intervention specialist (DIS) interview continuum leading to isolation in the network. Upon receiving a positive test, the index case is contacted for public health interview. The interview has two parts, an assessment of risk and partner elicitation for contact tracing by a DIS; the index can refuse to disclose contacts. If the index agrees to disclose contacts then the DIS conducting the interview elicits all contacts who may be the source of infection or who may have acquired the infection from the index (“source” and “spread”). A DIS then determines whether there is enough identifying information on contacts to initiate the tracing process and attempts to locate contacts who have sufficient information so that they may be offered testing. Contact tracing for an index case can end at any point in this process, resulting in an index case having zero traced contacts. This chart depicts the contact tracing steps, the end result of contact tracing for each HIV index case (N = 569), and where constructing the network added context to HIV index cases with zero of their own traced partners by showing where the HIV index cases with zero of their own traced contacts were identified as persons of interest in HIV or syphilis contact tracing investigations conducted during the same time period in the same geographic area. Nearly half (273/569, 48%) of HIV indexes had no located contacts during contact tracing. However, by linking HIV and syphilis investigations from the same time period and area, 25 of these 273 (9%) indexes were identified by another network member which adds context to the local HIV epidemic and permits public health personnel to better understand transmission patterns

Nearly one-quarter (128/569) of indexes did not disclose partners, though 10 (8%) of these 128 were then identified as a partner by another investigated HIV or syphilis case, so losses at that step in the DIS interview process contributed 118 rather than 128 of the 248 isolates (Fig. 3). In other words, 10 indexes who refused to disclose contacts had at least one network link and were not isolated because they were identified by another network member. The next largest loss was 56/569 (10%) indexes whose partners were investigated, though none could be located; however, 7 of 56 (13%) were identified by another network member. Along all of the partner services steps, linkages from other cases in the network “found” the highest proportion among indexes who only had partners without sufficient information to begin tracing (18%, 5/28).

Among all 248 indexes who remained isolated after allowing for linkages from other cases and contacts in the network, 54% (n = 134) did not disclose partners because they were not located or refused, 17% (n = 42) reported zero sexual partnerships, and 29% (n = 72) reported 1–50 partners (median = 2, IQR: 1–4) though none could be located (Fig. 3). However, 20/42 (48%) indexes who reported zero partners and were not identified by another network member were concurrently diagnosed with HIV and AIDS (late stage disease), and may not have had any partners to elicit in the 12 months prior to diagnosis (the interview window) (Hollingsworth et al. 2008).

Besides the 248 isolates, the remaining 321 indexes formed 220 discrete components of ≥ 2 people, most of which contained two people (n = 238 people across 119 dyads) or three people (n = 144 people across 48 components) when using both the HIV and syphilis investigations (Fig. 2a). The largest component included 320 persons (22% total network, 8% (46/569) indexes). Component size, dominant demographic characteristics, and member HIV status are shown in Tables 2 and 3. Among non-isolated indexes, 7 (1%) were in at least one triangle and 22 were in a *k*-core, comprising 21 (4%) in a 2-core and 1 (0.2%) in a 3-core.

**Table 2:** Dominant characteristics of sociosexual network components size 7 and smaller (n = 248 isolates and n = 201 components size 2–7), comprising 794 persons (54% total network)

Component size	Components (N)	People (N)	Number of indexes (mean)	Components at least half Black [n, (%)]	Components at least half female [n, (%)]	Components with transwomen (n)	Number of residents of Region 6 (mean)	Number known HIV + (mean)	Components with 1 syphilis edge [n, (%)]
1	248	248	1.0	149, (60)	52, (21)	1	1.0	1.0	0, (0)
2	119	238	1.1	88, (74)	64, (54)	1	1.8	1.3	0, (0)
3	48	144	1.2	39, (81)	14, (29)	0	2.6	1.8	2, (4)
4	20	80	1.1	15, (75)	6, (30)	0	3.1	1.8	1, (5)
5	5	25	1.2	3, (60)	0, (0)	0	2.8	2.6	1, (20)
6	4	24	1.3	3, (75)	0, (0)	0	3.8	2.8	0, (0)
7	5	35	1.4	4, (80)	1, (20)	0	4.8	3.0	0, (0)

**Table 3:** Demographic characteristics of 19 largest network components, comprising 676 persons (46% total network)

ID	People (N)	Total network (%)	Indexes (n)	White^a^ (%)	Black (%)	Hispanic (%)	Other (%)	Male^a^ (%)	Female (%)	Trans-women (%)	R6 residents (n)	Known HIV + (n)	Syphilis edges (n)
A	8	0.5	1	0	100	0	0	63	38	0	8	2	0
B	8	0.5	2	0	100	0	0	100	0	0	6	5	0
C	8	0.5	2	38	0	63	0	88	0	13	8	3	0
D	9	0.6	1	22	56	0	0	100	0	0	8	4	0
E	9	0.6	1	0	100	0	0	100	0	0	5	4	0
F	9	0.6	1	11	89	0	22	78	22	0	9	2	0
G	9	0.6	1	22	78	0	0	11	89	0	2	1	0
H	9	0.6	2	0	100	0	0	100	0	0	4	7	1
I	10	0.7	2	30	50	10	0	60	40	0	9	2	0
J	10	0.7	3	50	40	10	10	100	0	0	10	6	0
K	11	0.7	1	18	73	0	0	9	73	0	10	1	0
L	13	0.9	4	0	100	0	0	77	15	0	8	9	2
M	13	0.9	2	0	92	8	0	100	0	0	7	8	0
N	15	1.0	2	100	0	0	0	100	0	0	15	5	0
O	16	1.1	2	0	100	0	0	100	0	0	5	10	10
P	26	1.8	1	62	31	4	0	100	0	0	19	5	0
Q	81	5.5	9	1	90	0	2	94	5	0	39	28	37
R	92	6.3	18	8	85	1	7	93	4	1	47	45	36
S	320	21.8	46	38	51	5	5	97	0.1	0	164	136	137

^a^Ethnicity/race and gender do not sum to 100% for some components due to missing information

When restricting to the 321 non-isolated indexes, those who disclosed contacts which were initiated (n = 296) did not differ in terms of age, ethnicity/race, gender, or sexual preference from indexes who did not have any initiated contacts of their own but who were identified by another network member (n = 25). However, indexes who did not have any initiated contacts of their own were more likely to be in a large component. Though a similar proportion were in a component size 2 (10/25, 40%, vs. 115/296, 39%), indexes who did not have any initiated contacts were significantly more likely to be in one of the 3 largest components (44% vs. 21%, χ^2^ (1, N = 321) = 7.0, p < 0.01).

### Bridging of HIV by syphilis investigations

Ten (4%) of 220 components contained a mix of partnerships collected during syphilis investigations which encompassed overlapping people, with between 1 and 137 syphilis partnerships / edges (median = 1.5, IQR:1–36) included in the component (Tables 2 and 3). Median size of the 10 components containing HIV and syphilis investigation partnerships was 11 persons (IQR: 4–81, range: 3–320), including 2 HIV indexes (median, IQR:1–9, maximum = 46) and 0–22 syphilis indexes (median = 0, IQR: 0–2). Persons involved in syphilis investigations accounted for 11–63% of these components (mean = 32%, SD = 15%). The 3 largest of the 10 components (n = 81, 92, and 320 people) (Fig. 2b) included two or more HIV indexes bridged by partnerships elicited during syphilis investigations and would have appeared to be several smaller, separate components without the syphilis information (see component sized 92 in Fig. 2b for an example). In the component n = 320, several closed loops included a mix of HIV and syphilis investigation partnerships. Among 2012–2013 investigations contributing to these 10 components, 74 were indexes in HIV investigations only and 63 were indexes in a syphilis investigation (may or may not have been an HIV index as well). Cases who were only HIV indexes were more likely to not participate in contact disclosure (14%) compared to cases who were involved in syphilis only or syphilis and HIV investigations (3%, p = 0.04). Among indexes who did disclose contacts, there was no significant difference by infection in the average number of contacts per month across the period of interest.

### Post-study period HIV diagnoses

Of 221 first-degree partners of HIV index cases who were documented to be HIV-positive, 35 (16%) were diagnosed with HIV following the 2012–2013 investigation period during 2014–2018 (32 during 2014–2016 and 3 during 2017–2018) (Table 1). Half (17/35) had evidence of a negative HIV test result which was collected 2012–2013 and reported to the surveillance database, typically collected 2 + years prior to HIV diagnosis date (median 2.2 years, IQR:1.7–3.5, range:1.3–3.9 years). The other 18 did not have a record of a 2012–2013 HIV test result: 3 refused, 3 couldn’t be located at that time, 1 noted a current negative result though the laboratory result was not present in the database, 2 noted negative test results from prior to the study period which were not repeated, 5 no indication, and 4 had only the future positive test recorded. One in the last category was a sexual partner without current or recent negative HIV test results who was diagnosed in early 2014 as a result of partner tracing of an index case in this analysis.

Of the 201 network members who were part of a syphilis investigation and not first-degree partners to an HIV index case, 139 were initiated for partner services and not noted to be HIV-positive during the 2012–2013 study period (29 documented HIV negative and 110 unknown HIV status). Of these 139, 9 (6%) had evidence of 2014–2016 HIV diagnoses in the surveillance database and at least 4 (3%) were diagnosed in 2017 and 2018. All 13 were in the 3 largest components constructed from the 2012–2013 investigations, which were predominantly MSM and included both HIV and syphilis investigations.

## Discussion

Contact tracing is a key component of public health for infectious disease control of HIV and syphilis. Many challenges affect data quality and completeness during contact tracing and partner notification services, which can dampen service effectiveness. By incorporating information from multiple DIS investigations and examination of cases and their contacts in a more holistic way, sociosexual network analysis can mitigate some of the effects of unobserved partnerships underlying the HIV epidemic. Upon constructing the network, we were able to glean information about cases whose partners were not disclosed or located, which places those cases in a risk context related to the structure and composition of the components into which they were linked. That indexes who did not disclose partners but were identified by another network member were more likely to appear in larger components than people who did disclose located partners merits further investigation.

A median time of two years between negative and positive HIV tests among the HIV contacts who were later diagnosed is an indicator that behaviors sufficient for HIV acquisition continued even after there was an opportunity for public health intervention. Identifying the correlates of risk in a broader context can distinguish where preventive efforts should be directed. The overlap between HIV and syphilis in the larger components is consistent with prior research (Juher et al. 2017) and with clinical guidelines directing preventive HIV treatment (pre-exposure prophylaxis, or PrEP) evaluations toward people diagnosed with syphilis, further supported by the apparent subsequent acquisition of HIV in some of the syphilis network members. HIV and syphilis share at-risk populations and a primary mode of transmission. One limitation of the reportable disease mandate is that lab tests for HIV and syphilis with negative results are not reportable. Thus, we were not able to document negative HIV status for the majority of the second-degree syphilis investigation network members and some may have been HIV-positive during the study period but were not tested.

All 13 of the syphilis investigation members who tested positive for HIV after the study period were in large components with both HIV and syphilis circulating widely. This suggests that inclusion in one of these large components is an excellent indicator that pre-exposure prophylaxis (PrEP) to protect against HIV is warranted, particularly if the person has a recent sexually transmitted bacterial infection. Beyond an evaluation for PrEP, directing additional resources toward supporting an ongoing patient-provider relationship or subsidizing a prescription for PrEP for men who are part of these large components with circulating HIV and syphilis might have the benefit of averting cases, which can have a network-wide effect (Jenness et al. 2016).

As the largest components would have appeared to be smaller components without including information from the syphilis investigations, the inclusion of these edges revealed a more accurate picture of the underlying sociosexual network than would have been observed from the HIV investigations alone. Sociosexual networks provide an opportunity for intervention to reduce HIV transmission by adding context to public health contact tracing investigations so that limited resources can be directed toward areas of uncontrolled HIV or risk behaviors which have the potential for transmission.

## Data Availability

The datasets generated and/or analyzed are not publicly available. This analysis uses public health contact tracing and surveillance data, and is thus considered protected health information.

## Abbreviations

AHI: Acute HIV infection
AIDS: Acquired immunodeficiency syndrome
DIS: Disease investigation specialist
HIV: Human immunodeficiency virus
IQR: Interquartile range
MSM: Men who have sex with men
NC: North Carolina
PrEP: Pre-exposure prophylaxis
RNA: Ribonucleic acid
SD: Standard deviation
STD: Sexually transmitted disease
USA: United States
